# Resistance of *Ascochyta rabiei* isolates from chickpeas (*Cicer arietinum* L.) to fungicides

**DOI:** 10.1016/j.heliyon.2024.e35795

**Published:** 2024-08-03

**Authors:** Ali Endes, Amin Mohammed Yones, Sevim Atmaca, Muhidin Tahir, Mukaddes Kayim

**Affiliations:** a Department of Plant Protection, Faculty of Agriculture, Yozgat Bozok University, Yozgat, Turkey; b Department of Horticultural Sciences, College of Agriculture, Oda Bultum University, P.O. Box 226, Chiro, Ethiopia; c Department of Biology, College of Natural and Computational Sciences, Oda Bultum University, P.O. Box 226, Chiro, Ethiopia; d Department of Plant Protection, Faculty of Agriculture, Çukurova University, Sarıçam, Adana, Turkey

**Keywords:** *Ascochyta rabiei*, *Cicer arietinum*, Disease severity, Fungicide resistance

## Abstract

Ascochyta blight is a disease that causes significant yield losses in chickpea crops in Turkey under favorable environmental conditions. The fungal pathogen *Ascochyta rabiei* is the causative agent of this disease. The antifungal activity of previous fungicides against *A. rabiei* was not effective due to the heterothallic nature of the fungus. The aim of this study was to determine the sensitivity of *A. rabiei* to fungicides (25.2 g kgˉ^1^ boscalid + 12.8 g kgˉ^1^ pyraclostrobin; 50 % tebuconazole + 25 % trifloxystrobin; 62.5 g Lˉ^1^ propiconazole + 37.5 g Lˉ^1^ azoxystrobin; 80 % thiram; 80 % kükürt (sulphur); 80 % mancozeb; 80 % maneb) under *in vitro* and field conditions. Pure cultures of *A. rabiei* were isolated from infected chickpea plants collected in Boğazlayan, Sarıkaya, Sorgun, Merkez and Yerköy. A total of 14 *A. rabiei* isolates and 4 references were evaluated. The field test was conducted at Yozgat Bozok University, Yerköy Agricultural Application and Research Center Station. The trials began on March 14, 2021. The experimental area was divided into plots and the susceptible chickpea variety Sarı98 was used for the study. Two artificial inoculations were carried out approximately on the 40th and 80th days after sowing. Twenty-four hours after inoculation, the chickpea plants were sprayed with the fungicides Nativo® WG 75, Bellis®, Dikotan® M45 and Thiovit Jet® using a handheld sprayer. *In vitro* testing revealed that *A. rabiei* was resistant to kükürt (sulphur), thiram, maneb, and mancozeb. A field study showed that the percentage of *A. rabiei* isolates treated with the mancozeb fungicide was between 14 and 21 % of the control. Therefore, effective disease management strategies should include not only the use of fungicides, but also alternative approaches such as the use of resistant varieties. Moreover, the study focused on phenotypic resistance and suggests that future research should investigate the genetic and molecular mechanisms underlying *A. rabiei* resistance to enable better resistance management.

## 1 Introduction

Chickpea (*Cicer arietinum* L.) is one of the most important legumes in the world due to its good source of nutrients, including proteins, essential fatty acids, and medicinal value [[1], [2], [3]]. It also plays an important role in reducing poverty and hunger and maintaining ecosystem balance [4]. In addition to its nutritional value, chickpea improves soil fertility through its unique ability to biologically fix atmospheric nitrogen [5,6]. In addition, chickpea is one of the most widely grown crops among edible grain legumes in Turkey. For example, Turkey produced 0.47 million tons of chickpeas on 0.48 million hektares in 2021, ranking fourth in global chickpea production after India, Australia and Ethiopia [7]. In 2022, Yozgat produced 67,115 tons of chickpeas in an area of about 51,706 ha and is expected to rank 2nd in chickpea production in Turkey (TUIK, unpublished data from 2022). Although chickpea are grown in a large area of the country, the average chickpea productivity in Turkey (0.98 tons ha^−1^) is quite low compared to other chickpea producing countries worldwide (2.09 tons ha^−1^) [7]. Ascochyta blight was frequently observed in the chickpea growing areas of Yozgat; therefore, fungicides have been used extensively to control this disease. However, the effectiveness of these fungicides can be compromised by the development of resistant *A. rabiei* [8,9].

The low chickpeas yield in Turkey can be explained by biotic factors, especially fungal pathogens [10,11]. *Aschocyta rabiei* is a fungal pathogen that causes ascochyta disease in chickpea [12,13]. It is a disease that occurs in all chickpea is growing regions in Turkey and worldwide [11]. This disease limits chickpea production and causes severe crop losses of up to 100 % under favorable conditions [14]. Therefore, producers do not achieve sufficient yields every year [15]. Symptoms of this disease include leaf wilt, leaf lesions and stem damage, leading to stem breakage and pod lesions and ultimately seed disease [[16], [17], [18]]. The most common damage is caused by stem breakage and pod infections [19]. Fungal lesions are circular or elongated leaflets surrounded by reddish-brown outlines [20,21]. On green pods, pycnidia are concentric rings with circular lesions surrounded by black lines [18,22].

Globally, fungicides classified as protective or systemic are recommended to be sprayed on the seeds and green parts of chickpea infected with ascochyta disease [6,23]. Fungicides with protective effects should be used before infection, whereas fungicides with systemic effects can be used after infection [24]. Three groups of systemic fungicides, namely demethylation inhibitors (DMIs), external quinone inhibitors (QoIs) and succinate dehydrogenase inhibitors (SDHIs), are commonly used to protect chickpea plants from ascochyta blight [25]. During critical flowering and pod filling periods, systemic application of fungicides against ascochyta blight is usually recommended under favorable conditions [25]. There is evidence that seed treatment with systemic fungicides such as strobilurins increases the resistance of young plants [6]. In addition, the combination of thiabendazole with other fungicides can prevent the spread of the disease through contaminated seeds [24].

Although chickpea growers use fungicide uncontrollably and intensively for fear of losing their crops due to ascochyta disease, there is no clear fungicide spraying program against this disease in Turkey [26]. In addition, a previous study found that the effectiveness of fungicides that farmers use against different genotypes of *A. rabiei* may decrease or disappear over time due to the heterothallic nature of this fungus [5,24], resulting in ascochyta blight disease the most difficult to control [[27], [28], [29], [30]].

Understanding the resistance of *Ascochyta rabiei* to fungicides is crucial for developing effective disease control strategies. The aim of this study was to gain valuable insights into the current status of fungicide resistance in the region by assessing the extent and potential mechanisims of resistance and ultimately contribute to the development of sustainable disease management practices in chickpea cultivation. It is crucial to determine the resistance status of *A. rabiei* to commonly used fungicides to develop effective disease management strategies. Therefore, the aim of this study was to investigate the resistance of *A. rabiei* to fungicides under *in vitro* conditions and to determine the effects of commonly used fungicides on *A. rabiei* under field conditions.

## 2 Materials and methods

### 2.1 Isolation of A. rabiei

Chickpea tissue with disease symptoms was collected in Boğazlayan, Sarıkaya, Sorgun, Merkez and Yerköy districts. Samples were surface sterilized by soaking in 70 % ethanol for 1 min, followed by soaking in 1 % sodium hypochlorite solution for 2 min, and then rinsed three times in sterile distilled water. These sterilized tissue fragments were placed on potato dextrose agar (PDA) plates containing streptomycin to inhibit bacterial growth. Plates were incubated for 5–7 days at 22–25 °C with a 12-h light/dark cycle. Emerging fungal colonies were transferred to new PDA plates to obtain pure cultures. The purified isolates were identified based on their morphological characteristics such as spore size, shape, color, and colony appearance. Pure cultures of *A. rabiei* isolates collected from Boğazlayan (YBUAr1), Sarıkaya (YBUAr2), Sorgun (YBUAr6), Merkez (YBUAr7), and Yerköy (YBUAr9). In addition, four pathotypes of *A. rabiei* were obtained from the Department of Biology, Faculty of Science, Gaziantep University and used as references. A total of 14 *A. rabiei* isolates and 4 reference pathotypes were used in the study.

## 3 Descriptions of plant material and fungicides used in the study

The plant material used in the field study was the chickpea variety Sarı98, which is known to be susceptible to all pathotypes of *A. rabiei* [11]. The fungicides used in the current study are listed in Table 1.

**Table 1:** Descriptions of the fungicides used in the current study.

Active ingredient	Trade name	Company	Formulation
50 % Tebuconazole + % 25 Trifloxystrobin	Nativo®	Bayer	WG
25.2 g kgˉ^1^ Boscalid +12.8 g kgˉ^1^ Pyraclostrobin	Bellis®	Basf	WG
62.5 g Lˉ^1^ Propiconazole +37.5 g Lˉ^1^ Azoxystrobin	Altis® Premier	Hektaş	SC
80 % Kükürt	Thiovit Jet®	Syngenta	WG
80 % Thiram	Pomarsol Forte®	Bayer	WP
80%Mancozeb	Dikotan ®M45	Koruma Klor	WP
80 % Maneb	Dikotan ®M22	Koruma Klor	WP

WG = Water Dispersible Granule; SC = Suspension Concentrate; WP = Wettable Powder.

### 3.1 Fungal inoculation

All fungal isolates were incubated on chickpea seed dextrose agar (CSMDA: chickpea seed 40 g, dextrose 20 g and agar 15 gL^-1^) for 3 weeks at 22 ± 1 °C under a 12–h fluorescent cold white light photoperiod [18]. To harvest the conidia, 10 mL of sterile distilled water containing 0.01 % Tween 20 was added to each Petri dish and the conidia were inserted into the water using sterile scalpel. The mycelium was then thoroughly pounded with a mortar and pestle and the resulting suspension was filtered through a two layers of cheesecloth. The conidia concentrations were determined using a hemocytometer and adjusted to 2 × 10^5^ pycnidiospores mL^−1^ [11].

### 3.2 In vitro fungicide test

The list of fungicides used under *in vitro* conditions for this study is described in Table 1. Potato dextrose agar (PDA) was used to determine the degree of mycelial growth inhibition (EC_50_) of each fungicide [24]. To obtain the desired fungicide doses, dilutions were carried out with stock solutions prepared at high doses stock solutions. The stock solutions were prepared with doses of 1000, 100 and 10 ppm. Sterile distilled water was used to prepare all stock solutions and dilutions [29]. The dilution was carried out by adding the fungicide solution to PDA in flasks sterilized in an autoclave and cooled to 50 °C [21].

The medium containing the desired fungicide and the control (without fungicide) were poured in equal amounts into sterile Petri dishes and frozen for a while [31]. The Inoculations were made from 14-day-old pure cultures containing mycelia. Discs with a diameter of 4 mm, which were taken from the edges of the colonies of the experimental cultures using a cork borer, were inoculated into control Petri dishes with and without fungicide. During inoculation, care was taken to ensure that the fungal growth surfaces of the disks were in contact the medium, and one disk was placed in each Petri dish. After inoculation, the Petri dishes were kept in an incubator at 22 ± 1 °C without light for 21 days [21]. The experiments were performed in four replicates and repeated once.

### 3.3 Effects of fungicides against ascochyta blight disease under field conditions

A field test was conducted at Yozgat Bozok University, Yerköy Agricultural Application and Research Center Station (39°38ʹ58.03ʹN and 34°29ʹ39.07ʹE). The trial began on March 14, 2021, with the preparation of the experimental areas and the sowing of Sarı98 chickpea varieties. Two artificial inoculations were carried out on approximately day 40 (April 23, 2021) and day 80 (June 2, 2021) after sowing. Twenty-four hours after inoculation, the fungicides Nativo® WG 75, Bellis®, Dikotan®M45 and ThiovitJet® were applied to the chickpea plants using a handheld sprayer according to the manufacturer's instructions. The field trial was conducted in a randomized block design with three replicates [5 fungicides control (sterile water, 0); 14 *A. rabiei* isolates; 1 susceptible chickpea variety; 3 replicates; 5 × 9 × 1 × 3 × 3 = 135 plots]. One block consisted of 45 plots. Each plot consisted of three rows, each 3 m long. Chickpea planting was carried out at a seed density of 50 cm between rows and 10 cm between plants. A distance of 150 cm gap was left between the individual plots. The resistance of *A. rabiei* isolates to fungicides was assessed at the pod stage of the chickpea crop [32]. A scale of 1–9 was used to determine the ascochyta blight disease severity [19,33]. Disease severity index was determined using the following formula:

(1) Diseaseseverity(%)=[Ʃ(n×V)/Z×N]×100

Where n is the number of samples corresponding to different disease degrees on the scale, V is the scale value, Z is the highest, and N is the total number of samples observed.

### 3.4 Data analysis

Mycelial growth rates at fungicide doses were subjected to probit analysis and ED_50_ values (dose inhibiting mycelial growth by 50 %) were determined. The obtained data were subjected to analysis of variance (ANOVA) using SPSS version 25.0 statistical software. Means were separated using Tukey's HSD multiple comparison test (ɑ = 5 %) at the 5 % significance level.

## 4 Results and discussion

### 4.1 *In vitro* evaluation of fungicides against *A. rabiei*

**Fig. 1:**
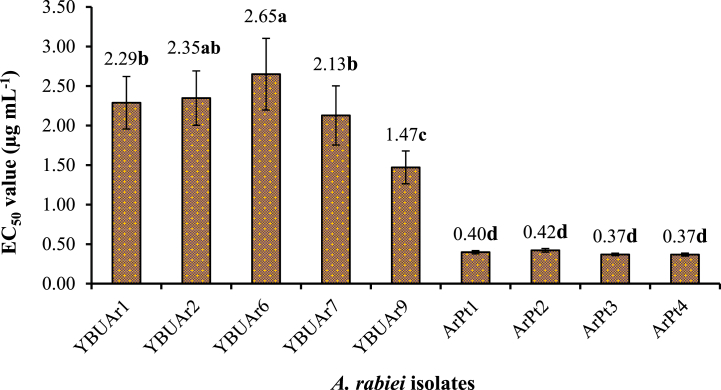
Effect of *A. rabiei* isolates on the EC_50_ value. The bars represent the means of four replicates of each fungicide for the EC_50_ value of each isolate. Vertical lines represent standard errors. Bars with the same letter are not significantly different according to Tukey's HSD (ɑ = 0.05) multiple comparison test.

In the *in vitro* evaluation of *A. rabiei* resistance to fungicides, analysis of EC50 values (μg mL^−1^) showed significant effects of both fungicides and isolate interactions and fungicides (Table 2). The main effect of *A. rabiei* on EC50 values differed significantly between the isolates (p < 0.0001) (Fig. 1).

**Table 2:** Analysis of variance of various fungicides’ effects on the EC50 values of *A. rabiei* isolates.

Source of variation	DF	SS	MS	F	Pr > F
Corrected model	62	657.353^a^	10.6025	64.9316	0.0001^b^
Intercept	1	481.427	481.427	2948.36	0.0001^b^
Isolates	8	220.255	27.5318	168.61	0.0001^b^
Fungicide	6	154.723	25.7871	157.925	0.0001^b^
Isolates*fungicide	48	282.376	5.88283	36.0276	0.0001^b^
Error	189	30.8612	0.16329		
Total	252	1169.64			
Corrected total	251	688.214			

DF = degrees of freedom; SS = sum of squares; MS = mean squares.

a = R square = 0.960 (adjusted R square = 0.946).

b significant at *p* < 0.05 difference.

*A. rabiei* isolates were divided into four classes based on their response to fungicides. Isolates ArPt1, ArPt2, ArPt3, and ArPt4, originating from areas outside Yozgat, showed the highest sensitivity to fungicides. In contrast, five *A. rabiei* isolates collected from different chickpea growing areas of Yozgat province were resistant to fungicides (Fig. 1). Among these strains, YBUAr6 from Sorgun region exhibited the highest level resistance. The resistance of YBUAr1, YBUAr2 and YBUAr7 from Bogazlayan and Merkez regions was moderate, while that of YBUAr9 was the lowest.

However, the resistance level of YBUAr2 strains obtained from the Sarıkaya region increased from moderate to high. In addition, the EC50 values of the *A. rabiei* isolates are listed in Table 3. Similarly, Chang et al. [34] reported the resistance levels of *A. rabiei* to chlorothalonil, mancozeb and pyraclostrobin and reported that 49 of 66 *A. rabiei* isolates developed resistance to one or more fungicides and that 26 of 37 isolates became resistant to chlorothalonil and pyraclostrobin spore germination. Furthermore, Arıcı and Evsen [26] reported that mancozeb was the least effective fungicide for the mycelial growth of *A. rabiei*. The results of this study are consistent with those of previous studies [24,31].

**Table 3:** The EC_50_ values of *Ascochyta rabiei* (μg mL^−1^).

Fungicide	Isolate								
	YBUAr1	YBUAr2	YBUAr6	YBUAr7	YBUAr9	ArPt1	ArPt2	ArPt3	ArPt4
Nativo®	0.467a	0.476a	0.329a	1.150b	0.551ab	0.375b	0.396bce	0.348ab	0.329b
Bellis®	0.400a	0.333a	0.091a	0.057a	0.110a	0.206a	0.222a	0.294a	0.208a
Altis® Premier	3.420bce	2.280bce	0.348a	0.354ab	0.689ab	0.342b	0.348ab	0.295a	0.321b
Thiovit Jet®	4.580c	4.705d	5.166d	0.515ab	1.774bce	0.386b	0.542c	0.334ab	0.326b
Pomarsol Forte®	3.994c	4.079d	2.487b	4.103c	2.076c	0.425b	0.419bce	0.440b	0.439c
Dikotan ®M45	2.350b	3.626cd	6.370e	5.036d	2.185c	0.53c	0.546c	0.425b	0.510c
Dikotan ®M22	0.805a	0.931ab	3.753c	3.677c	2.910c	0.51c	0.47bce	0.45b	0.442c
Mean	2.288	2.347	2.649	2.127	1.471	0.398	0.422	0.370	0.368

Values with the same letters are not significantly different (*p* = 0.05) according to Tukey's HSD test.

On the other hand, when 1.0 μg mL^−1^ was used as the discriminating concentration for the response of *A. rabiei* to fungicides, all isolates were still sensitive to the fungicide Bellis®. Nativo® for YBUAr7; Altis® Primer for YBUAr1 and YBUAr2; Thiovit Jet® for the YBUAr1, YBUAr2, YBUAr6 and YBUAr9; Dikotan ®M22 for YBUAr6, YBUAr7 and YBUAr9; and *A. rabiei* for the fungicides Dikotan ®M45 and Pomarsol Forte®.

The ability of fungicides to disrupt various parts and functions of mycelial growth in fungi has been demonstrated in several studies. For example, the boscalid disrupts fungal respiration by binding to the succinate dehydrogenase enzyme complex, distrusting the electron transport chain and ultimately leading to fungal cell death [35]. Similarly, pyraclostrobin acts by binding to the Qo site of the cytochrome *bc*1 complex, thereby impending electron transfer in the mitochondrial respiratory chain and causing a collapse in cellular energy production [36]. Tebuconazole targets fungal sterol biosynthesis by inhibiting lanosterol 14α-demethylase [37]. Trifloxystrobin disrupts fungal respiration by binding to the Qo site of the cytochrome *bc*1 complex, thereby impeding electron transfer and causing a failure in energy production [32]. Propiconazole blocks lanosterol 14α-demethylase, and hinders fungal sterol biosynthesis [32]. Azoxystrobin, a strobilurin fungicide similar to pyraclostrobin and trifloxystrobin, disrupts fungal respiration by binding to the Qo site of the cytochrome *bc*1 complex, leading to the breakdown of the mitochondrial respiratory chain [38]. On the other hand, mancozeb, a multisite contact fungicide, disrupts various fungal cellular processes, including enzyme function, respiration and cell division, by binding to the sulfhydryl groups of proteins [39]. Finally, maneb, a dithiocarbamate fungicide, inhibits fungal respiration by interfering succinate dehydrogenase activity [40].

Consistent with the present findings, Demirci et al. [33] found that the EC50 values of fungicides such as mancozeb (Penncozeb), maneb (Hektaneb M − 22), and thiram (Pomarsol Forte®) were particularly high, which is probably due to the extensive and intensive use of these fungicides. Our study also observed differences in the resistance level of *A. rabiei* isolates to the fungicides Dikotan®M45, Dikotan®M22, Thiovit Jet® and Pomarsol Forte®, which are widely used in Yozgat Province due to their affordability and easy of availability.

**Fig. 2:**
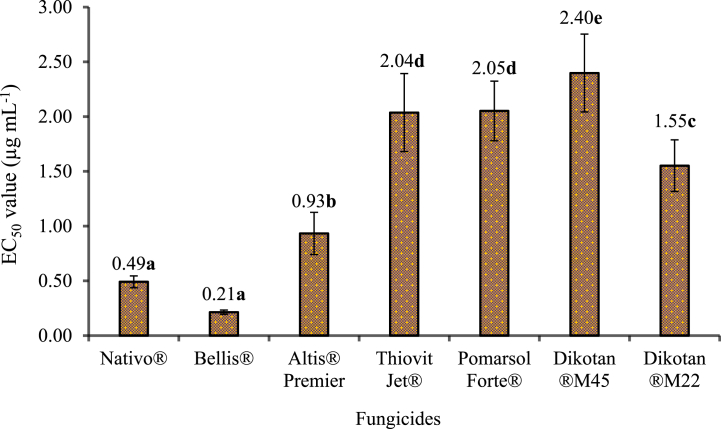
The main effect of fungicides on the EC_50_ value. The bars represent the means of four replicates of each isolate for the EC_50_ value of each fungicide. Vertical lines represent standard errors. Bars with the same letter are not significantly different according to Tukey's HSD (ɑ = 0.05) multiple comparison test.

In the main effect analysis of fungicides on EC50 values, a significant difference was observed between fungicides (p < 0.0001). Fungicides were divided into 5 classes based on their EC50 values. Bellis® and Nativo® found to be the most effective fungicides in inhibiting mycelial growth of all isolates, followed by Altis® Premier, Dikotan®M22, Thiovit Jet® and Pomarsol Forte®, while Dikotan® M45 was the least effective(Fig. 2).

### 4.2 Effects of fungicides against A. rabiei under field conditions

Evaluation of treatments under field conditions revealed significant interaction between the isolates, fungicides, and isolate + fungicide. The main effect of fungicides on disease severity was significant (p < 0.0001) (Table 4).

**Table 4:** Analysis of variance of different fungicides effect on the disease severity of *A. rabiei* isolates under field conditions.

Source of variation	DF	SS	MS	F	Pr > F
Corrected model	44	29465.672^a^	669.674	311.481	0.0001^b^
Intercept	1	144584.757	144584.757	67249.792	0.0001^b^
Fungicide	4	24617.123	6154.281	2862.502	0.0001^b^
Isolate	8	2965.147	370.643	172.395	0.0001^b^
Fungicide^b^isolate	32	1883.402	58.856	27.375	0.0001^b^
Error	90	193.497	2.150		
Total	135	174243.926			
Corrected total	134	29659.169			

DF = degrees of freedom; SS = sum of squares; MS = mean squares.

a = R square = 0.960 (adjusted R square = 0.946).

b significant at *p* < 0.05 difference.

The average disease severity of control plants without foliar fungicide application was 56.8 %. Bellis® was most effective in reducing disease severity with an average reduction of 65.5 %, resulting in a calculated average disease severity of 19.6 % (Table 5). Nativo® was the second most effective fungicide (60.5 %), followed by Thiovit Jet® (51.9 %) and Dikotan®M45 (33.8 %).

**Table 5:** Effects of different fungicides on ascochyta blight disease severity under field conditions.

Isolate	Control (Distilled water)	Bellis®	Dikotan ®M45	Thiovit Jet®	Nativo®
Disease severity (%)					
YBUAr1	55.5	20.9	46.5	33.5	25.1
YBUAr2	59.1	22.3	46.2	34.7	26.9
YBUAr6	58.5	24.7	50.0	35.3	27.7
YBUAr7	55.5	21.2	45.9	33.2	25.4
YBUAr9	53.8	18.5	44.3	29.8	24.5
ArPt1	55.4	17.4	27.0	19.5	18.9
ArPt2	58.8	16.7	24.7	19.9	18.0
ArPt3	59.3	17.2	26.3	20.2	17.7
ArPt4	55.5	17.7	26.7	19.5	17.4
Main effect of the fungicide	56.8e	19.6a	37.5d	27.3c	22.4b
**Efficacy of the fungicides (%)**					
YBUAr1	─	62.3	16.2	39.7	54.8
YBUAr2	─	62.3	21.8	41.3	54.5
YBUAr6	─	57.8	14.4	39.5	52.7
YBUAr7	─	61.9	17.2	40.2	54.2
YBUAr9	─	65.6	17.6	44.7	54.5
ArPt1	─	68.6	51.3	64.9	65.8
ArPt2	─	71.7	57.9	66.2	69.3
ArPt3	─	71.1	55.7	65.9	70.2
ArPt4	─	68.1	51.9	64.9	68.7
Mean	─	65.5	33.8	51.9	60.5

Values with the same letters are not significantly different (*P* = 0.05) according to Tukey's HSD test.

**Fig. 3:**
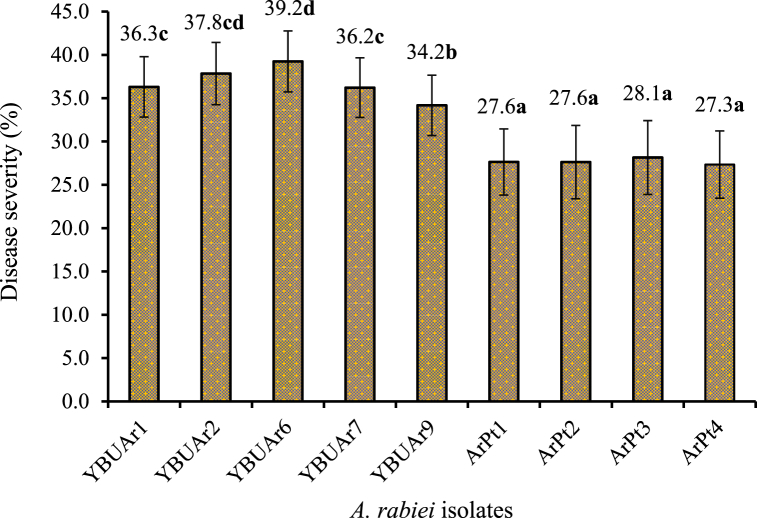
The main effect of *A. rabiei* isolates on the disease severity rate. The bars represent the means of four replicates of each isolate for the EC_50_ value of each fungicide. Vertical lines represent standard errors. Bars with the same letter are not significantly different according to Tukey's HSD (ɑ = 0.05) multiple comparison test.

In field studies, statistically significant differences btween fungicide responses to ascochyta blight disease severity were observed (Table 4), leading to their classification into four groups (Fig. 3). When the threshold for distinguishing disease severity was set at 30 %, ArPt1, ArPt2, ArPt3 and ArPt4 showed the highest sensitivity to fungicides. Consistent with findings from *in vitro* studies, YBUAr6 was the most resistant antifungal in field studies. However, YBUAr1, YBUAr2 and YBUAr7 showed medium resistance, while YBUAr9 showed the lowest resistance. Previous studies have reported the use of integrated control strategies to combat aschochyta blight [26,41,42]. The use of resistant varieties, crop rotation, healthy seeds and multiple applications of multiple fungicides in combination significantly reduce disease severity and increase yield [23,[43], [44], [45], [46]].

Spraying effective seed fungicides reduces inoculum emergence and disease spread to cleaned areas. If the field is contaminated or there are infected areas nearby, spraying fungicides alone is sometimes not enough to control *A. rabiei*. Several chemicals have been tested for this active ingredient, and some of them are partially effective [47]. Lichtenzveig et al. [25] reported that fungicides such as maneb, tebuconazole or difenoconazole can be used to control the pathogen, but the contribution of resistant varieties to disease control is much greater than that of chemical control methods.

In contrast, Lonergan et al. [48] reported the susceptibility of *Ascochyta* isolates to boscalid, fluxapyroxad and prothioconazole showed that they had minimal effects on disease distribution and severity. In contrast, Nene [49] documented ongoing efforts since 1931 to develop resistant varieties to ascochyta blight by selecting naturally resistant varieties. Similarly, in Canada, demonstration studies with moderately resistant varieties reported that ascochyta disease intensity reached 45 % under field conditions, while in Israel, 35 % of infections were recorded under similar circumstances [50,51]. Planting disease-free, asymptomatic seeds during sowing in the field reduces disease severity and increases disease resistance.

## 5 Conclusions and recommendations

The emergence of *A. rabiei* isolates resistant to commonly used fungicides represent a significant obstacle to efficient disease management in chickpea cultivation in Yozgat Province. The results of this investigation demonstrated the need for alternative and integrated disease management approaches. These approaches may include the introduction of resistant chickpea variety, the implementation of cultural practices such as crop rotation and sanitation, and the development of novel fungicides with different modes of action. Furthermore, this study highlights the importance of continuous surveillance and monitoring of *A. rabiei* populations to detect changes in resistance patterns. Such insights can help farmers and researchers make informed decisions regarding fungicide selection and apllication, ultimately reducing the risk of resistance emergence. The current study focused on phenotypic resistance but did not address the genetic or molecular mechanisms underlying *A. rabiei* resistance to the tested fungicides, suggesting that future research should investigate these mechanisms to provide deeper insights into resistance management.

**CRediT authorship contribution statement**

**Ali Endes** contributed to conceptualization, formal analysis, funding acquisition, investigation, and project administration; **Amin Mohammed Yones** was involved in data curation, formal analysis, and visualization and contributed to the original draft as well as review and editing. **Sevim Atmaca** contributed to the conceptualization, data curation, investigation, and writing of the original draft. **Muhidin Tahir and Mukaddes KAYIM** were responsible for the original draft, review, and editing of the language and sequence of the manuscript.

## Funding

This study was supported by the 10.13039/501100006564Bozok University Project Coordination Application and Research Center, BAP unit with project number 6602c-ZF 18–231.

## Data availability statement

The datasets used during the current study are available from the corresponding author on reasonable request.

## Declaration of competing interest

The authors declare the following financial interests/personal relationships which may be considered as potential competing interests:Ali Endes reports financial support was provided by Yozgat 10.13039/501100006564Bozok University. Ali Endes reports a relationship with Yozgat Bozok University that includes: employment. If there are other authors, they declare that they have no known competing financial interests or personal relationships that could have appeared to influence the work reported in this paper.
